# Population characteristics of pathogenic *Escherichia coli* in puerperal metritis of dairy cows in Ningxia region of China: a systemic taxa distribution of virulence factors and drug resistance genes

**DOI:** 10.3389/fmicb.2024.1364373

**Published:** 2024-04-17

**Authors:** Shihao Wei, Baolong Ding, Guiqin Wang, Shuangyan Luo, Hongxi Zhao, Xingang Dan

**Affiliations:** College of Animal Science and Technology, Ningxia University, Yinchuan, China

**Keywords:** dairy cows, drug resistance gene, drug sensitivity, *Escherichia coli*, puerperal metritis, virulence gene

## Abstract

*Escherichia coli* (*E. coli*) is closely associated with the occurrence of puerperal metritis in dairy cows. *E. coli* carries some the virulence and multi-drug resistant genes, which pose a serious threat to the health of postpartum cows. In this study, *E. coli* was isolated and identified from the uterine contents of postpartum cows with puerperal metritis in the Ningxia region of China, and its phylogenetic subgroups were determined. Meanwhile, virulence and drug resistance genes carried by *E. coli* and drug sensitivity were detected, and the characteristics of virulence and drug resistance genes distribution in *E. coli* phylogroups were further analyzed. The results showed that the isolation rate of *E. coli* in puerperal metritis samples was 95.2%. *E. coli* was mainly divided into phylogroups B2 and D, followed by groups A and B1, and was more connected to O157:H7, O169:H4, and ECC-1470 type strains. The virulence genes were mainly dominated by *ompF* (100%), *traT* (100%), *fimH* (97%), *papC* (96%), *csgA* (95%), *Ang43* (93.9%), and *ompC* (93%), and the resistance genes were dominated by *TEM* (99%), *tetA* (71.7%), *aac(3)II* (66.7%), and *cmlA* (53.5%). Additionally, it was observed that the virulence and resistance gene phenotypes could be divided into two subgroups, with subgroup B2 and D having the highest distributions. Drug sensitivity tests also revealed that the *E. coli* was most sensitive to the fluoroquinolones enrofloxacin, followed by macrolides, aminoglycosides, tetracyclines, β-lactams, peptides and sulfonamides, and least sensitive to lincosamides. These results imply that pathogenic *E. coli*, which induces puerperal metritis of dairy cows in the Ningxia region of China, primarily belongs to the group B2 and D, contains multiple virulence and drug resistance genes, Moreover, *E. coli* has evolved resistance to several drugs including penicillin, lincomycin, cotrimoxazole, and streptomycin. It will offer specific guidelines reference for the prevention and treatment of puerperal metritis in dairy cows with *E. coli* infections in the Ningxia region of China.

## 1 Introduction

Puerperal metritis is a frequent reproductive disorder in dairy cows following parturition, which can result in substantial uterine mucosa damage and incomplete uterine involution, lowering the reproduction and production performance while raising treatment costs and culling rates. Studies have demonstrated that dairy cows with puerperal metritis can delay next pregnancy for up to 95 days, and their failure rate to conceive is 3.8 times higher than that of healthy cows (Piccardi et al., 2016; Ehsanollah et al., 2021). Bacteria are the main cause of puerperal metritis in dairy cows, of which *E. coli*, *Cryptobacterium septicum*, *Clostridium necrophorum* and *Prevotella melanogaster* have been considered to be the main pathogens and might impact the endometrium and ovarian function (Bicalho et al., 2012). It was reported that *E. coli* appeared in the uterus within a few days of postpartum cows, followed by C. *necrophorum* and *Cryptobacterium septicum* (Jeon and Galvao, 2018). Numerous studies have revealed that *E. coli* was one of the most important pathogenic bacteria causing postpartum uterine infections in dairy cows (Ma et al., 2018; Schouten et al., 2023). Though the random amplification of polymorphic DNA and 16S rRNA gene sequencing of uterine contents and vaginal secretions of dairy cows with puerperal metritis, Wang et al. (2013) demonstrated that *E. coli* was predominant in both uterus and vagina. Further studies have revealed that differentially expressed proteins of *E. coli* induced inflammatory responses in the uterus via various pathways involved in cell proliferation, apoptosis, oxidative stress, and cell metabolism (Piras et al., 2017; Najafi et al., 2022).

*Escherichia coli* populations are typically subdivided into four main phylogenetic taxa including A, B1, B2, and D groups (Shamsi et al., 2017; Aguirre-Sanchez et al., 2022). Based on different virulence determinants and pathogenic characteristics, *E. coli* strains can be classified into commensal *E. coli*, enteropathogenic *E. coli* (EPEC), and extraintestinal pathogenic *E. coli* (ExPEC) (Biran and Ron, 2018). ExPEC, mainly belonging to taxa B2 and D, are associated with mucosal infections in multiple tissues and promote inflammatory responses (Van et al., 2008; Yamamura et al., 2022). Among the six species of ExPEC that have been identified, endometrial pathogenic *E. coli* (EnPEC) is the primary parenteral pathogenic *E. coli* causing uterine infections in postpartum cows (Bicalho et al., 2010; Sarowska et al., 2019). Additionally, the pathogenicity of *E. coli* is primarily dependent on the strain serotype and the kinds of virulence factors (VFs) carried by *E. coli* (Raeispour and Ranjbar, 2018). Recent studies have demonstrated that the development of metritis is mainly associated with the gene expression of ExPEC virulence factors (Kasse et al., 2016; Raheel et al., 2020). The main virulence factors of *E. coli* associated with puerperal metritis in dairy cows are *fimH*, *astA*, *kpsMII*, and *hlyA*. These factors are primarily carried on phages, plasmids, or pathogenic islands (PAIs), and they can be transferred horizontally between different microbial strains (Huddleston, 2014; Pakbin et al., 2021). Malahlela et al. (2022) found that the virulence genes of *E. coli* type O157 were mainly *stx1*, *stx2*, *eaeA* and *hlyA*, which usually encoded toxins and adhesins causing mild or bloody diarrhea and hemorrhagic colitis in animals. Furthermore, Ban et al. (2015) observed that the virulence genes of *E. coli* type O169:H41 were mainly dominated by CFs, CS6, and CS8 colonization factors, which mediated the inflammatory response.

Furthermore, the inefficient treatment of uterine infections caused by ExPEC and the emergence of multi-drug resistance in pathogenic bacteria are associated with the type of drug-resistance genes carried by the strains. Existing strains carrying TEM-type β-lactams are relatively resistant to penicillin and broad-spectrum cephalosporins. Furthermore, most resistance genes can be transferred between different serotype strains of *E. coli* by plasmids, transposons, and integrons, which induce the emergence of multi-drug resistant strains (Ban et al., 2015). However, the drug resistance genes carried by *E. coli* strains may vary considerably attributed to geographical differences.

The Ningxia region is a highly concentrated dairy farming area in China, which is also an area with a high incidence of puerperal metritis. Our previous study demonstrated that the pathogenic bacteria causing puerperal metritis in dairy cows in the Ningxia region were mainly *E. coli* (unpublished data). However, to date, the distribution of *E. coli* populations in the uterine contents of dairy cows with puerperal metritis in this region remains elusive. Meanwhile, the virulence factors and drug resistance genes carried by *E. coli* are also unknown, which poses many challenges to for the prevention and treatment of puerperal metritis induced by *E. coli* in dairy cows. Therefore, in this study, we isolated and identified *E. coli* from the uterine contents of puerperal metritis in dairy cows in the Ningxia region of China and further performed the phylogenetic subgroup and homology analyses of *E. coli* in the region. Furthermore, the major virulence genes and drug resistance genes carried by *E. coli* were detected, and the distribution pattern of virulence factors and drug resistance genes of *E. coli* in its phylogenetic subgroups were analyzed. Additionally, the drug susceptibility analysis of *E. coli* in this region was conducted. This may provide some references for the prevention and control of puerperal metritis in dairy cows in this region.

## 2 Materials and methods

### 2.1 Ethics statement

All animal experiments in this study were reviewed and approved by the Animal Welfare Committee of Ningxia University (License No. NXUC20220906). All experimental methods were performed in accordance with the Regulations on the Management of Laboratory Animals (Revised 2021) regarding animal care and animal experiments.

### 2.2 Sample source and collection

Forty-two samples were collected from the uterine contents of cows suffering from metritis at 7–10 d postpartum from June to September 2022 in five different areas of Ningxia, including Wuzhong City (Field A), Yinchuan City (Field B), Shizuishan City (Field C), Pingluo County (Field D) and Lingwu City (Field E). Neosporin was used to clean the vulva of dairy cows before the samples were collected by a homemade sterile sampling device, which ensures effective collection of uterine contents and prevents microbial contamination of the vulva and vagina. The extracted samples were injected into sterile EP tubes and stored in an incubator at 4°C and then transported back to the laboratory for subsequent experiments.

### 2.3 Diagnosis of puerperal metritis in dairy cows

The diagnosis of puerperal metritis was conducted by a skilled veterinarian. Depressed, dehydrated, anorexic (fasting), shortness of breath, decreased milk production, tachycardia, decreased rumination, enlarged uterus on rectal examination. Cows with body temperature above 39.5°C and foul-smelling uterine contents were diagnosed with puerperal metritis (Lomb et al., 2018; Figueiredo et al., 2021).

### 2.4 Isolation and identification of *Escherichia coli*

The collected uterine contents were inoculated on EMB solid medium after 10-fold dilution with saline. Subsequently, monoclonal colonies were selected to be inoculated in TSB liquid medium. Next, the cultured bacterial fluid was inoculated again on EMB solid medium for purification culture. After numerous purifications, homogenous colony matching the colony’s morphology, size, and color formed on the solid medium. Further, typical colonies were picked and smeared for Gram staining followed by preliminary identification by morphology. Furthermore，single colonies were inoculated into TSB liquid medium for expanded culture. Then, bacterial DNA was extracted using the bacterial genomic DNA extraction kit, and bacterial 16S rDNA PCR amplification was conducted using bacterial universal primers. Amplification products recovered and purified after electrophoresis were sent to Shanghai Bioengineering Company for sequencing. Finally, the sequences were subjected to BLAST with those of the NCBI database, and the final determination of bacterial species was made. Universal primers for PCR amplification: F: AGAGTTTGATCCTGGCTCAG; R: TACGGCTACCTTGTTACGACTT. PCR reaction system comprised the following: 12.5 μL Premix TaqTM (TaKaRa TaqTM Version 2.0 plus dye), 1 μL template and 1 μL upstream and downstream primers, the concentration of each primer was 0.08 mΜ, and 9.5 μL ddH2O. PCR conditions were as follows: pre-denaturation at 94°C for 5 min; 30 cycles of denaturation 94°C for 30s, annealing at 55°C for 45 s, and extension at 72°Cfor 90s; extension at 72°C for 10 min.

### 2.5 Evolutionary analysis of *Escherichia coli*

Following 16S rDNA PCR amplification and sequencing, the obtained sequences were compared in the NCBI database, and the sequences of identified *E. coli* strains originating from different regions were selected as references. The homology relationship of strains was analyzed by using MEGA11 software.

### 2.6 Phylogenetic subgroups of *Escherichia coli*

Phylogenetic clustering was performed using multiple PCR targeting DNA fragments of genes chuA, yjaA, and TspE4-C2 to distinguish the four phylogenetic clusters A, B1, B2, and D. The specific phylogenetic clustering methods are indicated in Table 1 (Clermont et al., 2013; Nowrouzian et al., 2019). The primers of related gene fragments are depicted in Table 2. The 50 μL PCR reaction system comprised the following: 25 μL Premix TaqTM (TaKaRa TaqTM Version 2.0 plus dye), 2 μL template, 1 μL upstream and downstream primers, add ddH_2_O to 50 μL. Amplification conditions was as follows: pre-denaturation at 94°C for 5 min; 30 cycles of denaturation 94°C for 30s, annealing at 58°C for 45 s, and extension at 72°Cfor 90s; extension at 72°C for 10 min. Subsequently, *E. coli* was categorized based on the band size after electrophoresis.

**Table 1:** Phylogenetic grouping methods for *E. coli.*

System subgroups	Genes		
	*chuA*	*yiaA*	*TspE4-C2*
A	−	+/−	−
B1	−	+/−	+
B2	+	+	+/−
D	+	−	+/−

“+” in the table indicates a positive band, “−” indicates no band.

**Table 2:** PCR primers for phylogenetic identification of *E. coli.*

Genes	Primer sequences (5′-3′)	Annealing temperature°C	PCR fragment size(bp)	Primer source
*chuA*	(F)GACGAACCAACGGTCAGGAT (R)TGCCGCCAGTACCAAAGACA	58	279	Gonzalez et al. (2020)
*yiaA*	(F)TGAAGTGTCAGGAGACGCTG (R)ATGGAGAATGCGTTCCTCAAC	58	211	
*TspE4-C2*	(F)GAGTAATGTCGGGGCATTCA (R)CGCGCCAACAAAGTATTACG	58	152	

### 2.7 Detection of *Escherichia coli* virulence gene

The virulence factors carried by *E. coli* in the uterus of dairy cows in the Ningxia region were determined by amplifying virulence factor genes encoding ExPEC. PCR was used to detect virulence factor genes of each strain, and the reaction procedure and conditions were the same as those described in Method 2.4. PCR amplification primers and annealing temperatures are indicated in Table 3.

**Table 3:** PCR primers for *E. coli* virulence factors identification.

Type	Genes	Primer sequences (5′-3′)	Annealing temperature°C	PCR fragment size(bp)	Primer source
Pilus	*fimH*	(F)TGTTACGTCCTGTAGAAAGCCC (R)AAAACTGCCTGGCACAGCAATT	63	508	Gonzalez et al. (2020)
	*papC*	(F)GACGGCTGTACTGCAGGGTGTGGCG (R)TATCCTTTCTGCAGGGATGCAATA	50	380	
	*F17A*	(F)GCAGAAAATTCAATTTATCCTTGG (R)CTGATAAGCGATGGTGTAATTAAC	57	537	Bertin et al. (1996)
	*F41*	(F)GCATCAGCGGCAGTATCT (R)GTCCCTAGCTCAGTATTATCACCT	50	380	Gonzalez et al. (2020)
	*F5*	(F)TATTATCTTAGGTGGTATGG (R)GGTATCCTTTAGCAGCAGTATTTC	50	314	
	*csgA*	(F)ACTCTGACTTGACTATTACC (R)AGATGCAGTCTGGTCAAC	55	200	
Adhesins	*sfa*	(F)CTCCGGAGAACTGGGTGCATCTTAC (R)CGGAGGAGTAATTACAAACCTGGCA	63	410	
	*Afa*	(F)GGCAGAGGGCCGGCAACAGGC (R)CCCGTAACGCGCCAGCATCTC	63	559	
Protein	*ompF*	(F)TTTCCAAGGGTAACGGTGAA (R)CCATCAACCAGACCAAAGAAG	56	382	Washizaki et al. (2016)
	*ompC*	(F)CCGGTACCTAAAAAAGCAAATAAAGGCA (R)CCAAGCTTTGTACGCTGAAAACAATG	62	1,104	Zhang et al. (2021)
	*Eae*	(F)GGAACGGCAGAGGTTAATCTGCAG (R)GGCGCTCATCATAGTCTTTC	55	775	Gonzalez et al. (2020)
	*iutA*	(F)GGCTGGACATCATGGGAACTGG (R)CGTCGGGAACGGGTAGAATCG	68	301	
	*irp2*	(F)TTCCTTCAGTCGCCTGTTA (R)CAAGCCCGACATACTCAATCT	57	301	Paton and Paton (1998)
	*agn43*	(F)CTGGAAACCGGTCTGCCCTT (R)CCTGAACGCCCAGGGTGATA	58	433	Gonzalez et al. (2020)
Poison	*astA*	(F)GCTAATGTTGGCAATTTTTATTTCTGTA (R)AGGATTACAACAAAGTTCACAGCAGTAA	50	190	
	*stx2*	(F)GGCACTGTCTGAAACTGCTCC (R)TCGCCAGTTATCTGACATTCTG	60	255	Monroy-Perez et al. (2020)
	*LT1*	(F)AAACAAAACAAGTGGCG (R)GTTGTTATATAGGTTCCTAGC	50	711	Saez-Lopez et al. (2016)
Hemolysin	*hlyA*	(F)AACAAGGATAAGCACTGTTCTGGCT (R)ACCATATAAGCGGTCATTCCCGTCA	63	1,177	Gonzalez et al. (2020)
Poison Island	*ECs3703*	(F)TTGACATCATCAATCACCAATG (R)TCAATGTTGGACCGAATGTG	54	693	Jeon et al. (2015)
Other	*CnfL1*	(F)AAGATGGAGTTTCCTATGCAGGAG (R)CATTCAGAGTCCTGCCCTCATTATT	57	498	Monroy-Perez et al. (2020)
	*kpsMT2*	(F)GCGCATTTGCTGATACTGTTG (R)CATCCAGACGATAAGCATGAGCA	63	272	Gonzalez et al. (2020)
	*fyuA*	(F)TGATTAACCCCGCGACGGGAA (R)CGCAGTAGGCACGATGTTGTA	63	880	
	*traT*	(F)GGTGTGGTGCGATGAGCACAG (R)CACGGTTCAGCCATCCCTGAG	63	290	

### 2.8 Detection of drug resistance genes in *Escherichia coli*

Drug resistance analysis was performed by amplifying the resistance genes. PCR was used to detect virulence factor genes of each strain, and the reaction procedure and conditions were the same as those described in Method 2.4. PCR amplification primers and annealing temperatures are indicated in Table 4.

**Table 4:** PCR primers for *E. coli* drug-resistance genes detection.

Resistance	Genes	Primer sequences (5′-3′)	Annealing temperature°C	PCR fragment size(bp)	Primer source
Beta-lactams	*TEM*	(F) CATTTCCGTGTCGCCCTTATTC (R) CGTTCATCCATAGTTGCCTGAC	56	800	Monroy-Perez et al. (2020)
	*CITM*	(F) TGGCCAGAACTGACAGGCAAA (R) TTTCTCCTGAACGTGGCTGGC	60	462	
Chloramphenicol	*cmlA*	(F) CCGCCACGGTGTTGTTGTTATC (R) CACCTTGCCTGCCCATCATTAG	59	698	
Tetracycline	*tetA*	(F) GGTTCACTCGAACGACGTCA (R) CTGTCCGACAAGTTGCATGA	56	557	
Quinolone	*qnrA*	(F) GGGTATGGATATTATTGATAAAG (R) CTAATCCGGCAGCACTATTTA	50	670	
Sulfamethoxazole	*Sul1*	(F) TTCGGCATTCTGAATCTCAC (R) ATGATCTAACCCTCGGTCTC	52	882	
Trimethoprim	*dfrA1*	(F) GGAGTGCCAAAGGTGAACAGC (R) GAGGCGAAGTCTTGGGTAAAAAC	58	367	
Aminoglycoside	*aac(3)II*	(F) ACTGTGATGGGATACGCGTC (R) CTCCGTCAGCGTTTCAGCTA	57	237	

### 2.9 Drug susceptibility test

The susceptibility test of *E. coli* was done with 16 kinds of antibiotic susceptibility test papers commonly used in the clinic tests. Following diluting the concentration of bacteria solution with TSB liquid medium, absorbance was detected using a spectrophotometer. Absorbance value was in the range 0.08–0.1 by adjusting the concentration of bacteria solution, indicating that the number of bacteria in 0.5 McGallowi standard bacteria solution was about 1 × 10^8^ ~ 2 × 10^8^ (McLaughlin et al., 2012). After adjusting the concentration, the bacteria solution was evenly distributed on the plate with a disposable sterilized cotton swab. When the Agar surface was slightly dry, we used sterile forceps to attach the sensitive paper to the Agar surface, with four sheets of sensitive paper per plate. The petri dishes were then placed upside down in the incubator for ≥18 h. The results of drug susceptibility tests were analyzed per the standards of the American Committee for Clinical Laboratory Standardization (NCCLS). The results were divided into sensitive type (sensitive type, S), intermediate type (I) and resistant type (R) based on the diameter of the inhibition zone. The test was conducted in triplicate.

### 2.10 Statistical analysis

The phylogenetic evolutionary tree of *E. coli* strains was created using MEGA11 software to examine the homology relationship of the *E. coli* stains from each region. Origin software was used to construct stacked histograms and matrix plots to analyze the phylogenetic taxon subgroups of *E. coli* strains in each region and the distribution of *E. coli* virulence genes and drug resistance genes in the phylogenetic taxa.

## 3 Results

### 3.1 Isolation and identification of *Escherichia coli*

**Figure 1:**
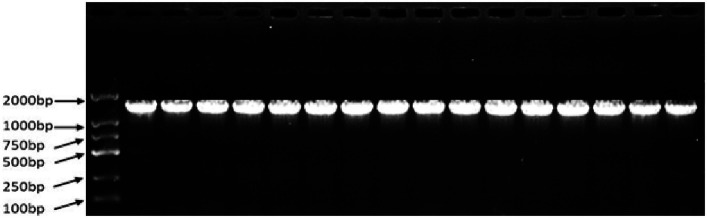
PCR amplification results of strains.

Following the isolation and identification of 42 samples, 99 strains of *E. coli* were finally obtained. *E. coli* colonies exhibited round, raised, and smooth surface colonies with metallic luster or black pigment production on EMB agar. Preliminary identification of bacteria was done using Gram staining, which revealed that Gram-negative bacteria were red-stained with short rods or rods with blunt rounded ends. PCR results revealed that the fragment size of amplification products was 1,500 bp (partly presented in Figure 1). Eventually, 99 strains were identified as *E. coli* following comparison of sequencing results with NCBI sequences.

### 3.2 Homology analysis of *Escherichia coli* strains

**Figure 2:**
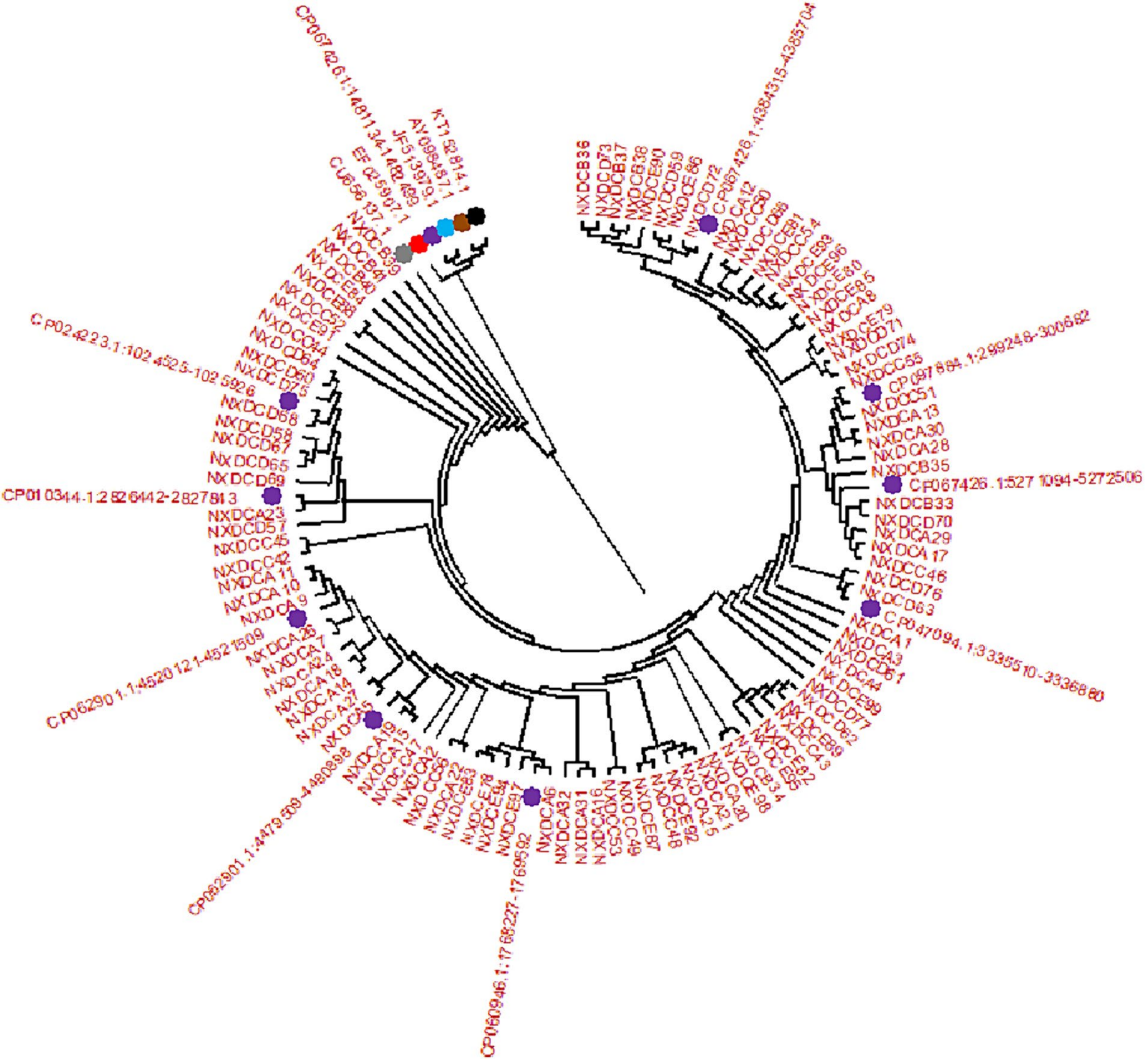
Phylogenetic tree created by *E. coli* 16S rDNA PCR amplification and sequencing. There are 99 isolate samples of 16S rDNA target sequences, corresponding to the sequences of (NXDCA1-A32), (NXDCB33-B41), (NXDCC42-C56), (NXDCD57-D77), and (NXDCE78-E99). There are 15 16S rDNA reference sequences, and the different color represent the reference sequences of different origins and types, including bovine uterus: KT152814.1, avian origin: AY098487.1, porcine origin: JF513979.1,sheep origin: EF025907.1, human origin: CU656137.1, and 10 classics strains of different types of *E. coli* CP067426.1 (O22:H8), CP024223.1 (O169:H41), CP010344.1 (ECC-1470), two types of CP062901 (O152:H23), CP060946.1 (O157:H7), CP047094.1 (Salmonella sp), two types of CP067426 (O22:H8), and CP097884.1 (K-12).

SeqMan and EditSeq were used for sequence analysis and splicing of 99 *E. coli* strains, and phylogenetic tree was constructed using these sequences with MEGA11. According to the findings, all strains developed two main branches, as presented in Figure 2. Among these, 99 target sequences were on the same large branch as that of human CU656137.1 and nine typical representative strains of different types of *E. coli* including CP024223.1, CP010344.1, CP062901 (both types), CP060946.1, CP047094.1, CP067426 (both types), and CP097884.1. Specifically, the *E. coli* strain from farm A in the Wuzhong area was on the same branch as that of CP062901.1. The *E. coli* strain from farm B in Yinchuan was on the same branch as that of CP067426.1 and CU656137.1 of human origin. The strain of *E. coli* from farm C in Huinong District was on the same branch as that of CP097784.1. The strain of *E. coli* from farm D in the Pingluo area was on the same branch as that of CP067426.1, CP047094.1, CP010344.1, and CP024223.1. The *E. coli* strain from farm E in Lingwu was on the same branch as that of CP060946.1.

### 3.3 Phylogenetic grouping of *Escherichia coli* inducing puerperal metritis

**Figure 3:**
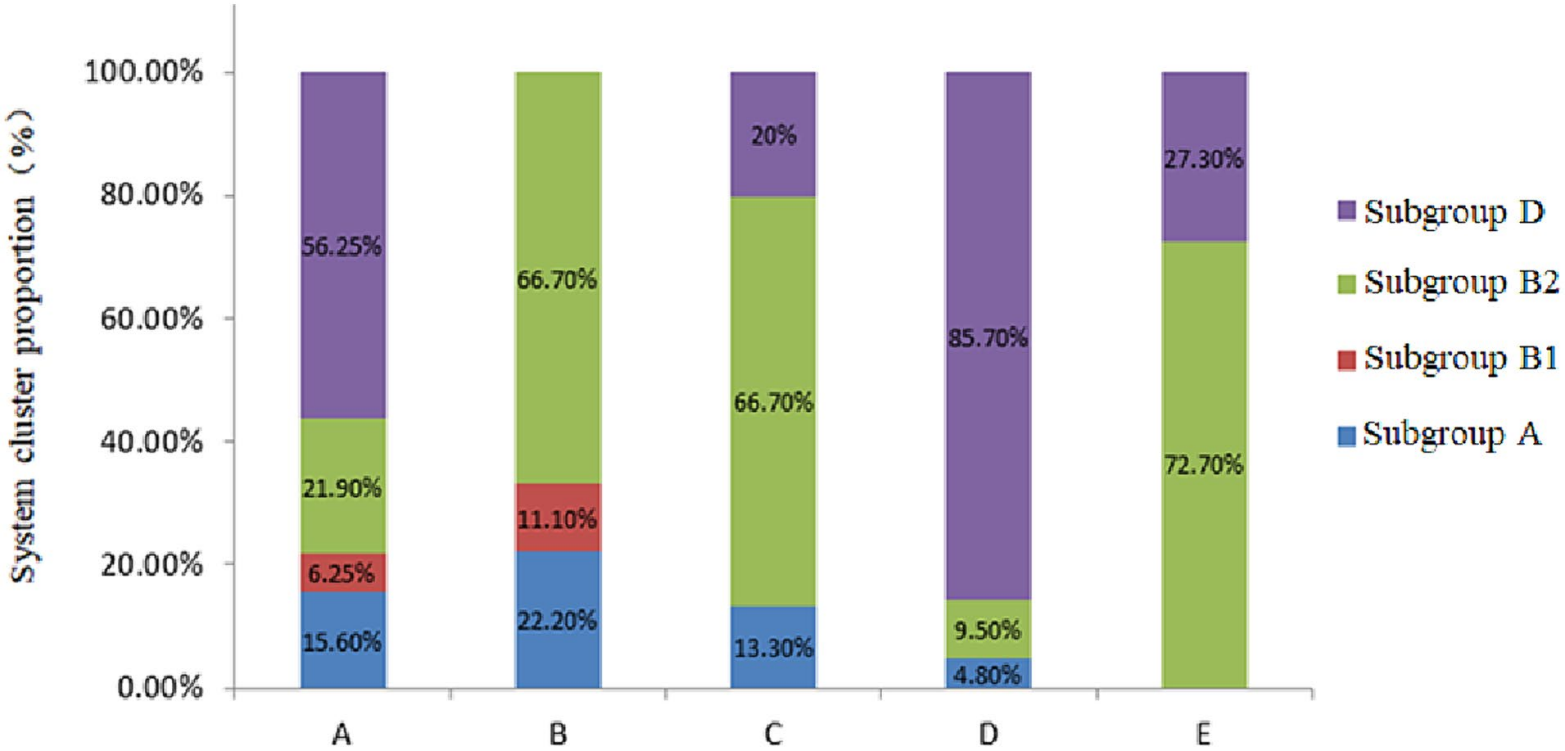
Phylogenetic subgroups of *E. coli* strains from different regions.

The four phylogenetic subgroups A, B1, B2, and D were distinguished using multiplex PCR (Figure 3). The results revealed that the Wuzhong (Farm A) area, which has four phylogenetic subgroups A, B1, B2 and D, was the most abundant in *E. coli* group categories. *E. coli* isolates from Yinchuan (Farm B) were mainly divided into subgroups A, B1, and B2. *E. coli* isolated from Huinong District (Farm C) and Pingluo (Farm D) were mainly distributed in subgroups A, B2, and D. Furthermore, *E. coli* isolated from Lingwu (Farm E) were distributed only in subgroups B2 and D. These results revealed that pathogenic *E. coli* from dairy cows with puerperal metritis in Ningxia primarily belonged to subgroups B2 (41.4%) and D (45.5%) followed by subgroups A (10.1%) and B1 (3.03%).

### 3.4 Analysis of *Escherichia coli* virulence factor

The findings of the *E. coli* virulence factor analysis are displayed in Table 5. The highest detection rate of virulence genes was 100% for both *ompF* and *traT*, and followed by *fimH* (97%), *papC* (96%), *csgA* (95%), *Ang43* (93.9%), *ompC* (93%), *iutA* (87.9%), *ECs3703* (81.8%), *irp2* (78.8%), *Afa* (64.5%), *fyuA* (59.6%), *stx2* (38.4%), *F17A* (27.3%), *KpsMTII* (21.2%), and *hlyA* (19.2%). However, the detection rates of *F14*, *F5*, *sfa*, *eae*, *astA*, *LT1*, and *Cnf1* were all zero. These results indicated the comparatively high abundance of virulence genes carried by ExPEC in this region, which were mainly dominated by *ompF*, *traT*, *fimH*, *papC*, a*csgA*, *Ang43*, and *ompC*.

**Table 5:** Analysis of *E. coli* virulence genes.

Type	Gene	Analysis of the virulence gene in different regions					Total number of strains *n* = 99(%)
		Farm A *n* = 32(%)	Farm B *n* = 9(%)	Farm C *n* = 15(%)	Farm D *n* = 21(%)	Farm E *n* = 22(%)	
Pilus	*fimH*	29 (90.6)	9 (100)	15 (100)	21 (100)	22 (100)	96 (97)
	*papC*	31 (97)	9 (100)	15 (100)	21 (100)	19 (86.4)	95 (96)
	*csgA*	31 (97)	8 (89)	15 (100)	21 (100)	19 (86.4)	94 (95)
	*F17A*	15 (47)	1 (11.1)	2 (13.3)	0 (0)	9 (41)	27 (27.3)
	*F41*	0 (0)	0 (0)	0 (0)	0 (0)	0 (0)	0 (0)
	*F5*	0 (0)	0 (0)	0 (0)	0 (0)	0 (0)	0 (0)
Adhesins	*sfa*	0 (0)	0 (0)	0 (0)	0 (0)	0 (0)	0 (0)
	*Afa*	18 (56.3)	5 (55.6)	12 (80)	12 (57.1)	17 (77.3)	64 (64.5)
Protein	*ompF*	32 (100)	9 (100)	15 (100)	21 (100)	22 (100)	99 (100)
	*ompC*	29 (90.6)	9 (100)	12 (80)	21 (100)	21 (95.5)	92 (93)
	*eae*	0 (0)	0 (0)	0 (0)	0 (0)	0 (0)	0 (0)
	*iutA*	25 (78.1)	5 (55.6)	14 (93.3)	21 (100)	22 (100)	87 (87.9)
	*Ang43*	31 (96.9)	7 (77.8)	15 (100)	20 (95.2)	20 (91)	93 (93.9)
	*irp2*	28 (87.5)	6 (66.7)	5 (33.3)	20 (95.2)	19 (86.4)	78 (78.8)
Poison	*astA*	0 (0)	0 (0)	0 (0)	0 (0)	0 (0)	0 (0)
	*stx2*	14 (43.8)	4 (44.4)	6 (40)	8 (38.1)	6 (27.3)	38 (38.4)
	*LT1*	0 (0)	0 (0)	0 (0)	0 (0)	0 (0)	0 (0)
Hemolysin	*hlyA*	4 (12.5)	0 (0)	2 (13.3)	4 (19)	9 (41)	19 (19.2)
Poison Island	*ECs3703*	29 (90.6)	5 (55.6)	13 (86.7)	13 (61.9)	21 (95.4)	81 (81.8)
Other	*KpsMTII*	2 (6.3)	0 (0)	0 (0)	8 (38.1)	11 (50)	21 (21.2)
	*fyuA*	24 (75)	1 (11.1)	5 (33.3)	14 (66.7)	15 (68.2)	59 (59.6)
	*traT*	32 (100)	9 (100)	15 (100)	21 (100)	22 (100)	100 (100)
	*Cnf1*	0 (0)	0 (0)	0 (0)	0 (0)	0 (0)	0 (0)

*n* refers to the number of *E. coli* strains isolated from each cattle farm, same below.

### 3.5 Analysis of *Escherichia coli* drug resistance gene

The findings of the *E. coli* drug resistance gene analysis are displayed in Table 6. *TEM* exhibited the highest detection rate of 99%, followed by *tetA* (71.7%), *aac(3)II* (66.7%), *cmlA* (53.5%), *qnrA* (48.5%), and *sul1* (26.3%) whereas *CITM* and *dfrA1* were detected at a detection rate of zero. There were significant differences in the detection rates of *cmlA*, *sul1*, *qnrA*, and *tetA* genes among the five regions, with the highest detection rate of 72.7% in farm E and the lowest of 37.5% in farm A for the *cmlA* gene. The highest detection rate of the *sul1* gene in Farm B and the lowest in Farm D was 9.5%. The highest detection rate of the *qnrA* gene was 100% in farm E, and the lowest detection rate of the *qnrA* gene was 12.5% in farm A. The highest detection rate of the *tetA* gene was 94% in farm A whereas that of the *tetA* gene in farm D was 57.1%. These results suggested that ExPEC was generally resistant to Beta-lactams and has varying degrees of resistance to other antibiotic drugs in the Ningxia region of China.

**Table 6:** Analysis of *E. coli* drug-resistance genes.

Types of antibiotics	Gene	Detection results of drug resistance genes in different regions					Total number of strains *n* = 99(%)
		FarmA *n* = 32(%)	Farm B *n* = 9(%)	Farm C *n* = 15(%)	Farm D *n* = 21(%)	Farm E (*n* = 22)%	
Chloramphenicol	*cmlA*	12 (37.5)	7 (77.8)	7 (46.7)	11 (52.4)	16 (72.7)	53 (53.5)
Aminoglycoside	*aac(3) II*	24 (75)	6 (66.7)	9 (60)	13 (61.90)	14 (63.6)	66 (66.7)
Sulfamethoxazole	*sul1*	8 (25)	5 (55.6)	2 (13.3)	2 (9.5)	9 (41)	26 (26.3)
β-lactams	*TEM*	31 (97)	9 (100)	15 (100)	21 (100)	22 (100)	98 (99)
	*CITM*	0 (0)	0 (0)	0 (0)	0 (0)	0 (0)	0 (0)
Quinolone	*qnrA*	4 (12.5)	6 (66.7)	8 (53.3)	8 (38.1)	22 (100)	48 (48.5)
Tetracycline	*tetA*	30 (94)	7 (77.8)	9 (60)	12 (57.1)	13 (59.1)	71 (71.7)
Trimethoprim	*dfrA1*	0 (0)	0 (0)	0 (0)	0 (0)	0 (0)	0 (0)

### 3.6 Distribution of virulence factors in *Escherichia coli* phylum taxa

**Figure 4:**
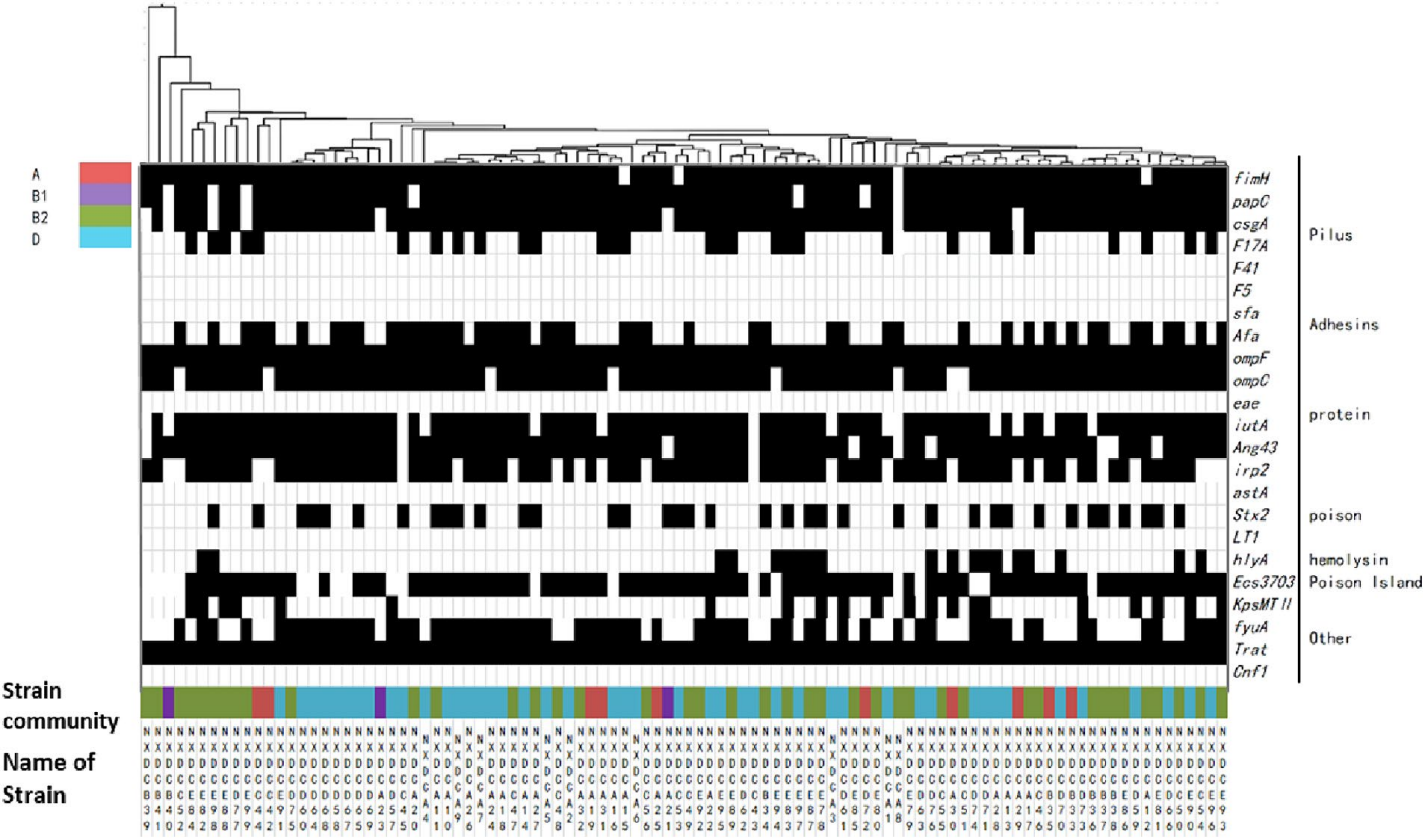
Matrix of distribution patterns of virulence factors in *E. coli* phylum taxa. Classification and clustering of 99 *E. coli* strains based on the association between virulence factors and phylogroups. The given genetic phenotypes are represented by black rectangles, and virulence factors and types are located on the right side of the graph. Systematic taxa are represented by different color codes, as indicated by the color labeling in the upper left corner of the figure above. The branches and names of the genes of the strains are indicated above and below, respectively.

The relationship between the virulence factors of the strains and their phylogenetic subgroups was analyzed, and the findings are displayed in Figure 4. The distributions of the genes *csgA*, *F17A*, *Afa*, *irp2*, *stx2*, *hlyA*, *KpsMTII*, *ECs3703*, and *fyuA* on the four phylogenetic taxa were significantly different. The detection rate of *irp2* was significantly lower in subgroup A than in subgroups B1, B2, and D. The detection rate of *fyuA* was significantly higher in subgroup D than in subgroups A, B1, and B2. Conclusively, the detection rates of virulence factors in subgroups B2 and D were significantly higher than those in subgroups A and B1. Additionally, the same virulence factors were observed in all of the closely associated isolates, and each strain contained at least four different virulence factors. These results indicated that the virulence factors were highly abundant in *E. coli* strains, which were mainly distributed in subgroups B2 and D in this region.

### 3.7 Distribution of drug resistance genes in phylum taxa of *Escherichia coli*

**Figure 5:**
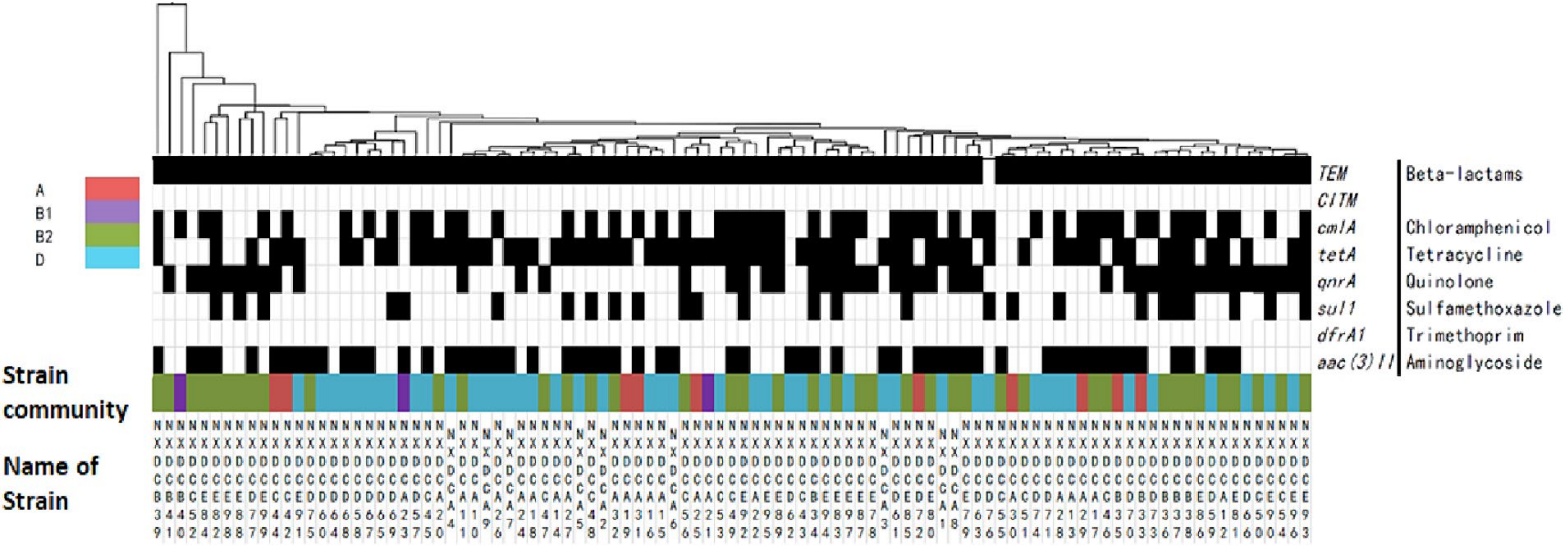
Matrix of the distribution patterns of drug resistance genes in *E. coli* phylogroups. Classification and clustering of 99 *E. coli* strains based on their drug resistance gene phenotypes in association with phylogroups and evolution. The identified genetic phenotypes are represented by black rectangles, and drug-resistance genes are located on the right side of the graph. Systematic taxa are indicated by different color coding, as depicted by the colors presented in the upper left corner of the figure above. The branches and names of the genes of the strains are displayed above and below, respectively.

The distribution of drug resistance genes in *E. coli* systemic subgroups are displayed in Figure 5. The distribution of *cmlA*, *sul1*, *aac(3)II*, and *qnrA* resistance genes on the four developmental taxa varied significantly. The detection rates of *cmlA* and *qnrA* in subgroup B1 were zero, which were significantly lower than those in subgroups A, B2, and D. The detection rate of *qnrA* was significantly higher in subgroup B2 than in subgroups B1, D, and A. The detection rate of *sul1* was significantly lower in subgroup D than in subgroups A, B1, and B2. The detection rate of *aac(3)II* was higher in subgroups A and D than in subgroups B1 and B2. Additionally, closely related strains exhibited nearly identical drug-resistance genes. These results suggested that the drug resistance genes carried by *E. coli* strains of different systemic groups were different, and the types of drug resistance genes carried by *E. coli* strains of groups B2 and D were much higher than those of groups A and B1.

### 3.8 Drug susceptibility analysis of *Escherichia coli*

Table 7 displays the drug susceptibility test results of 16 commonly used antibiotics for *E. coli*. Significant differences were observed in the sensitivity of *E. coli* to various drugs. The strains were most sensitive to fluoroquinolones enrofloxacin, followed by macrolides, aminoglycosides, tetracycline, β-lactams, polypeptides, and sulfonamides, and least sensitive to lincomycin. *E. coli* was resistant to penicillin, streptomycin, cotrimoxazole, and lincomycin but sensitive to cefotaxime, ofloxacin, and enrofloxacin.

**Table 7:** Drug sensitivity analysis of *E. coli* isolated different farms in different regions.

Category	Drug sensitive tablets	Results of drug susceptibility test of *E. coli* isolates in different regions																				Content ug/ tablet
		A				B				C				D				E				
Sensitivity		R	I	S	Drug resistance rate(%)	R	I	S	Drug resistance rate(%)	R	I	S	Drug resistance rate(%)	R	I	S	Drug resistance rate(%)	R	I	S	Drug resistance rate(%)	
β-lactams	*Amoxicillin*	12	14	6	37.5%	6	1	2	66.7%	11	1	3	73.3%	13	8	0	61.9%	15	5	7	68.2%	30
	*Penicillin*	9	11	12	28.1%	6	2	1	66.7%	7	8	0	46.7%	9	9	3	42.9%	6	9	7	27.3%	10
	*Cefotaxime*	16	4	12	50.0%	7	1	1	77.8%	13	1	1	86.7%	11	6	4	52.4%	15	6	1	68.2%	10
	*Cephalexin*	8	2	22	25.0%	5	4	0	55.6%	9	0	6	60.0%	17	0	4	81.0%	4	18	0	18.2%	30
Tetracycline	*Doxycycline*	5	11	16	15.6%	4	4	1	44.4%	6	6	3	40.0%	7	6	8	33.3%	6	8	10	27.3%	30
	*Tetracycline*	7	13	12	21.9%	3	3	3	33.3%	4	8	3	26.7%	7	10	4	33.3%	9	6	7	40.9%	30
Aminoglycosides	*Gentamicin*	4	5	23	12.5%	1	3	5	11.1%	4	6	5	26.7%	5	6	10	23.8%	4	9	9	18.2%	10
	*Kanamycin*	4	8	20	12.5%	3	3	3	33.3%	3	7	5	20.0%	7	9	5	33.3%	8	10	4	36.4%	10
	*Streptomycin*	5	6	21	15.6%	2	3	4	22.2%	3	6	6	20.0%	6	8	7	28.6%	6	5	11	27.3%	30
Macrolides	*Azithromycin*	5	7	20	15.6%	1	3	5	11.1%	2	6	7	13.3%	5	10	6	23.8%	5	8	9	22.7%	15
Fluoroquinolones	*Enrofloxacin*	1	3	28	3.1%	0	2	7	0%	1	3	11	6.7%	3	8	10	14.3%	3	6	13	13.6%	5
	*Ofloxacin*	3	6	23	9.4%	0	4	5	0%	2	6	7	13.3%	4	10	7	19.0%	5	5	12	22.7%	5
Peptides	*Polymyxin B*	21	10	1	65.6%	6	0	0	66.7%	9	3	3	60.0%	15	5	1	71.4%	17	5	0	77.3%	30
	*Vancomycin*	19	6	17	59.4%	7	2	0	77.8%	8	2	5	53.3%	12	3	6	57.1%	14	4	4	63.6%	30
Sulfonamides	*Cotrimoxazole*	14	16	2	43.8%	3	5	1	33.3%	5	8	2	33.3%	6	15	0	28.6%	10	12	0	45.4%	25
Lincoamides	*Lincomycin*	28	4	0	87.5%	7	2	0	77.8%	15	0	0	100%	19	2	0	90.5%	21	1	0	95.5%	2

## 4 Discussion

*Escherichia coli* has been considered as the most prevalent bacterial species in the cervix and vagina (Saez-Lopez et al., 2016). However, some studies on reproductive tract microbes have highlighted different perspectives on *E. coli*-induced puerperal metritis. Previous research has revealed that *E. coli* was nearly non-existent in the vaginal and uterine pathogenic microbial communities of dairy cows (Jeon et al., 2015; Bicalho et al., 2017; Jeon and Galvao, 2018). However, other studies have confirmed that *E. coli* played a decisive role in the development of puerperal metritis in dairy cows (Miller et al., 2007; Goldstone et al., 2014; Genis et al., 2016; Gonzalez et al., 2020). *Escherichia coli* of the uterus not only damaged the uterine mucosa but also increased the likelihood of subsequent uterine infection induced by *Cryptobacterium septicum*, *Bacillus necrophorum*, and other conditionally pathogenic bacteria in the early postpartum period, which was closely associated with the development of puerperal metritis in dairy cows (Bicalho et al., 2012). This study demonstrated that the isolation rate of *E. coli* strains from sample species was as high as 95.2%, making it the primary pathogen causing puerperal metritis of dairy cows in the Ningxia region of China.

*Escherichia coli* O157:H7 and *E. coli* O169:H41 have been reported to be highly pathogenic (Yang et al., 2017). Further study has observed that two strains belonged to enterohemorrhagic *E. coli* and caused bloody diarrhea and the potentially fatal hemolytic uremic syndrome (Razmi et al., 2020). Furthermore, *E. coli* O157:H7 and O169:H41, as important intestinal highly infectious pathogens, were strongly linked to the development of gastroenteritis (Jarocki et al., 2021). Meanwhile, the two *E. coli* induced an inflammatory response via the release of a Shiga-like toxin (Ten et al., 2017). Surprisingly, we also isolated strains with close affinity to O157:H7 and O169:H41 from the uterine fluid of dairy cows with puerperal metritis. It was speculated that intestinal pathogenic *E. coli* breaks through the intestinal barrier under certain conditions and reaches the uterus via blood or lymphatic circulation, causing uterine inflammation. Our hypothesis is substantially identical to the identified endogenous “intestinal-mammary gland” pathway transfer mechanism of the microorganisms (Luo et al., 2022; Zhao et al., 2022). Furthermore, certain bacteria that were first detected in the mammary gland have been discovered in animal digestive and reproductive tracts (Fernandez and Rodriguez, 2020). Additionally, research has demonstrated that some common pathogens causing mastitis in dairy cows, such as *Staphylococcus* and *Streptococcus*, were predisposed to infecting the mucosal layer of the digestive tract (Rodriguez, 2014). This further adds to the evidence that gastrointestinal and uterine microorganisms in dairy cows are related. Additionally, we discovered that *E. coli* ECC-1470 accounted for a larger proportion of isolated strains. A study has revealed that *E. coli* ECC-1470 isolated from the uterus of dairy cows with chronic puerperal metritis had become a frequent strain responsible for inflaming the uterus (Dogan et al., 2006). This is consistent with our findings.

Previous studies have revealed that ExPEC strains typically belonged to groups B2 and D of the phylogenetic taxa, which comprised numerous virulence determinants involved in parenteral infections (Van et al., 2008; Paniagua-Contreras et al., 2017). Another study has reported that strains from groups A and B1 were rarely associated with parenteral infections (Restieri et al., 2007). Our study discovered that subgroup B2 and D strains accounted for 41.4 and 45.5% in all strains isolated from dairy cows with puerperal metritis respectively, which carried numerous virulence genes associated with extraintestinal infections. However, subgroup A and B1 strains accounted for 10.1 and 3.03%, respectively, albeit the detection rates of virulence genes were low. These findings depict that the pathogenic *E. coli* strains were predominant in dairy cows with puerperal metritis in this region. However, Khawaskar et al. (2022) demonstrated that ExPEC strains of bovine origin were mainly abundant in subgroups A and C. This differed from our results. It was speculated that the different serotypes of the strains caused different distributions of the strains in the phylogenetic subgroups.

It is prominent that the pathogenicity of *E. coli* depends mainly on the type and amount of virulence factors. The common virulence factors are *fimH*, *ompF*, and *papC*, among which *fimH* and *papC* encode for type 1 hair genes (Schembri et al., 2000). It has been reported that the strains of *E. coli* carrying *fimH* increased the incidence of puerperal metritis in dairy cows by 4.6 times (Bicalho et al., 2012). Furthermore, *fimH* promotes bacterial colonization, invasion and biofilm formation, and enhances its pathogenicity (Floyd et al., 2015). The virulence factor *ompF*, a membrane protein of ExPEC, adheres to and invades host cells (de Pace et al., 2010). Also, the expression of ompF in *E. coli* strains has been reported to affect the susceptibility of the strain to antibiotic drugs (Huguet et al., 2013; Zhang et al., 2018). Khalifeh and Obaidat (2022) observed that 97 and 32% of the bovine strains carried *fimH* and *papC*, respectively, and these strains typically carried two or more virulence genes. Additionally, Mihailovskaya et al. (2022) reported that *fimH* (91.8%), *afa* (61.2%), *iutA* (44.9%), and *papC* (20.4%) were predominant in the virulence genes carried by ExPEC. In this study, we observed that the main virulence genes carried by *E. coli* were ompF (100%), *traT* (100%), *fimH* (97%), and *papC* (96%), and each strain contained multiple virulence genes. *TraT* has been reported to be more prevalent in *E. coli* isolated from urine and blood samples (Abd et al., 2020). Further studies have revealed that *traT* encodes resistance genes and increases the risk of infection via ColV plasmid transfer (Cha et al., 2016). In this study, we observed the detection rate of *traT* to be 100%. It was speculated that *E. coli* carrying the *traT* virulence gene migrates to the uterine via blood circulation, and *traT* is further horizontally transferred between strains with the help of plasmids or phages (Huddleston, 2014), indicating that uterine pathogenic *E. coli* also carries *traT* gene.

The long-term use of antibiotics has caused the emergence of multi-drug resistance in pathogenic bacteria. Taylor et al. (2021) demonstrated that triple cephalosporins had low susceptibility to *E. coli* strains in dairy cows with puerperal metritis. The emergence of pathogenic bacterial resistance genes seriously affects the effectiveness of antibiotic therapy. Furthermore, Goudarztalejerdi et al. (2022) observed that *tetA* had the highest detection rate of 68%, followed by *sul1* (63%), *dfrA1* (51%), and *TEM* (30%) in avian pathogenic *E. coli* strains. In this study, drug resistance genes analysis revealed that the highest detection rate of β-lactam-resistant *TEM* genes was 99%, followed by *tetA* (71.7%), *sul1* (26.3%), and *dfrA1* (0). The detection rates of *TEM* and *dfrA1* gene were significantly higher than those reported above. It was discovered that the types of resistance genes carried by *E. coli* strains were distinct since some resistance genes were transferred horizontally between strains with the help of plasmids, transposons, and integrons, among which *TEM* was the most prevalent (Styles et al., 2022). In this study, we observed that the *TEM* gene was present in almost all strains and the detection rate was 99%, which is consistent with aforementioned findings. Furthermore, previous studies have reported that strains carrying *TEM*-type β-lactams were typically resistant to both penicillin and broad-spectrum cephalosporins (Poirel et al., 2004; Jeong et al., 2018). We also discovered that strains carrying *TEM*-type β-lactams had low susceptibility to penicillin and significantly lower susceptibility to broad-spectrum cephalosporins such as cefotaxime and cefadroxil compared to other types of drugs, which was consistent with aforementioned findings.

Additionally, there is an association between phylogroups of *E. coli* strains and their distribution of virulence and drug resistance genes. Halaji et al. (2022) discovered that 28 Vfs of avian pathogenic *E. coli* strains belonged to subgroups B2 and D, and *ecA*, *fyuA*, *irp2*, and *kspMTII* were abundant, which was significantly higher than those in subgroups A and B1. Futhermore, Halaji et al. (2022) demonstrated that subgroups B2 and D were the major phylogenetic taxa of UPEC strains, and UPEC strains in subgroups B2 and D were more resistant than those in subgroups A and B1. Furthermore, both APEC and UPEC are subtypes of ExPEC, which indicates that ExPEC strains are almost entirely distributed in subgroups B2 and D. In this study, *E. coli* strains belonging to subgroups A and B1 harbored significantly fewer virulence factors and drug resistance genes than those belonging to phylogenetic taxa B2 and D, which is consistent with the findings above.

In this study, although we discovered pathogenic *E. coli* to be the predominant bacteria of dairy cows with puerperal metritis in the Ningxia region of China, we also observed that *E. coli* mainly belonged to groups B2 and D and carried several virulence genes and drug resistance genes in this region. However, further research is required to understand the processes by which virulence and drug resistance genes carried by *E. coli* regulate the development of puerperal metritis in dairy cows and the development of drug resistance in *E. coli*.

## 5 Conclusion

In the Ningxia region of China, the infection rate of puerperal metritis induced by *E. coli* in dairy cows was 95.2%, and the strains were mainly distributed in groups B2 and D, with close affinity to (O157:H7), (O169:H4), and (ECC-1470) type strains. Additionally, these *E. coli* mainly carried seven virulence genes including *ompF* (100%), *traT* (100%), *fimH* (97%), *papC* (96%), *csgA* (95%), *Ang43* (93.9%), and *ompC* (93%) and five drug resistance genes containing *TEM* (99%), *tetA* (71.7%), *aac(3)II* (66.7%), and *cmlA* (53.5%). Additionally, *E. coli* is resistant to penicillin, streptomycin, cotrimoxazole, and lincomycin, but responsive to cefadroxil, ofloxacin, and enrofloxacin. However, there is a further need to analyze the reasons for the development of drug resistance in these pathogens as well as to investigate the molecular mechanisms by which these bacteria develop drug resistance.

## Data availability statement

The data presented in the study are deposited in the NCBI repository, accession numbers PP455648 - PP455745.

## Ethics statement

The animal study was approved by Animal Welfare Committee of Ningxia University. The study was conducted in accordance with the local legislation and institutional requirements.

## Author contributions

SW: Writing – original draft, Conceptualization, Data curation, Formal analysis, Visualization. BD: Writing – original draft, Investigation, Methodology, Validation. GW: Writing – review & editing, Project administration. SL: Writing – original draft, Software. HZ: Writing – review & editing, Resources. XD: Writing – review & editing, Funding acquisition.
